# Multi-task deep learning-based radiomic nomogram for prognostic prediction in locoregionally advanced nasopharyngeal carcinoma

**DOI:** 10.1007/s00259-023-06399-7

**Published:** 2023-08-19

**Authors:** Bingxin Gu, Mingyuan Meng, Mingzhen Xu, David Dagan Feng, Lei Bi, Jinman Kim, Shaoli Song

**Affiliations:** 1 Department of Nuclear Medicine, Fudan University Shanghai Cancer Center, Shanghai, People’s Republic of China; 2 Department of Oncology, Shanghai Medical College, Fudan University, Shanghai, People’s Republic of China; 3 Center for Biomedical Imaging, Fudan University, Shanghai, People’s Republic of China; 4 Shanghai Engineering Research Center of Molecular Imaging Probes, Shanghai, People’s Republic of China; 5 Key Laboratory of Nuclear Physics and Ion-Beam Application (MOE), Fudan University, Shanghai, People’s Republic of China; 6 School of Computer Science, the University of Sydney, Sydney, Australia; 7 Institute of Translational Medicine, National Center for Translational Medicine, Shanghai Jiao Tong University, Shanghai, China

**Keywords:** Radiomics, Deep learning, Nasopharyngeal carcinoma, PET/CT, Survival prediction

## Abstract

**Purpose:**

Prognostic prediction is crucial to guide individual treatment for locoregionally advanced nasopharyngeal carcinoma (LA-NPC) patients. Recently, multi-task deep learning was explored for joint prognostic prediction and tumor segmentation in various cancers, resulting in promising performance. This study aims to evaluate the clinical value of multi-task deep learning for prognostic prediction in LA-NPC patients.

**Methods:**

A total of 886 LA-NPC patients acquired from two medical centers were enrolled including clinical data, [^18^F]FDG PET/CT images, and follow-up of progression-free survival (PFS). We adopted a deep multi-task survival model (DeepMTS) to jointly perform prognostic prediction (DeepMTS-Score) and tumor segmentation from FDG-PET/CT images. The DeepMTS-derived segmentation masks were leveraged to extract handcrafted radiomics features, which were also used for prognostic prediction (AutoRadio-Score). Finally, we developed a multi-task deep learning-based radiomic (MTDLR) nomogram by integrating DeepMTS-Score, AutoRadio-Score, and clinical data. Harrell's concordance indices (C-index) and time-independent receiver operating characteristic (ROC) analysis were used to evaluate the discriminative ability of the proposed MTDLR nomogram. For patient stratification, the PFS rates of high- and low-risk patients were calculated using Kaplan–Meier method and compared with the observed PFS probability.

**Results:**

Our MTDLR nomogram achieved C-index of 0.818 (95% confidence interval (CI): 0.785–0.851), 0.752 (95% CI: 0.638–0.865), and 0.717 (95% CI: 0.641–0.793) and area under curve (AUC) of 0.859 (95% CI: 0.822–0.895), 0.769 (95% CI: 0.642–0.896), and 0.730 (95% CI: 0.634–0.826) in the training, internal validation, and external validation cohorts, which showed a statistically significant improvement over conventional radiomic nomograms. Our nomogram also divided patients into significantly different high- and low-risk groups.

**Conclusion:**

Our study demonstrated that MTDLR nomogram can perform reliable and accurate prognostic prediction in LA-NPC patients, and also enabled better patient stratification, which could facilitate personalized treatment planning.

**Supplementary Information:**

The online version contains supplementary material available at 10.1007/s00259-023-06399-7.

## Introduction

Nasopharyngeal carcinoma (NPC) is an epithelial malignancy arising from the nasopharyngeal mucosal lining [1], with high prevalence rates in east and southeast Asia [2]. About 70%-80% of NPC patients are categorized as locoregionally advanced NPC (LA-NPC) (Tumor-Node-Metastasis (TNM) stage III or IVa) ﻿according to the 8th edition of American Joint Committee on Cancer (AJCC)/Union for International Cancer Control (UICC) staging system [3]. The primary therapeutic regimen for NPC is radiation therapy (RT) with or without chemotherapy due to its radiosensitivity [4]. However, despite the improvement in treatment, due to locoregional recurrences and distant metastasis, the 5-year survival rates of LA-NPC patients is still a persistent problem, usually ranging from 10 to 40% [5]. Under this circumstance, pretreatment prognosis is a major concern for LA-NPC patients, which is conducive to guide the individualized therapeutic regimen. Specifically, based on the pretreatment prognosis, patients could be stratified into different risk groups with different therapeutic regimens applied, and this has been reported to potentially improve the patients’ overall survival outcomes [6].

TNM staging system is widely used for prognostic prediction and patient stratification [7–9]. However, despite the fact that patients with the same TNM stage receive the same treatment, large variations in prognosis exists due to the heterogeneous nature of tumor microenvironment [10]. Image-derived biomarkers, such as the ﻿standardized uptake value (SUV) and metabolic tumor volume (MTV) derived from [^18^F]-fluorodeoxyglucose ([^18^F]FDG) positron emission tomography/computed tomography (PET/CT), can provide promising prognostic information for NPC [11, 12]. Nevertheless, these factors are limited in clinical practice as they are arduous to represent intra-tumor information such as tumor texture, intensity, heterogeneity, and morphology. Therefore, a reliable and accurate prognostic prediction model is needed to predict their progression-free survival (PFS), and to distinguish high-risk from low-risk patients. Such prediction will ultimately facilitate the formulation of therapeutic regimens and improve patients’ overall survival outcomes.

Radiomics is a widely recognized computational method for prognostic prediction, which extracts high-dimensional handcrafted features from medical images to characterize intra-tumor information and then models the relevance between the features and prognostic outcomes through statistical methods [13, 14]. ﻿Radiomics has been widely used for prognostic prediction in various cancers including NPC [15–17]. However, the extraction of radiomics features requires tumor segmentation masks as the guidance, which inevitably brings an additional segmentation step into the radiomics pipeline. In addition, radiomics features are extracted from the segmented regions, which are usually limited to primary and metastatic lesions [5, 18]. This suggests that the extracted radiomics features may have difficulties in representing the prognostic information outside of malignant lesions (e.g., adjacent tissue invasion). There have been attempts at leveraging lymph node segmentation for radiomics analysis [19–21]. However, lymph node segmentation is intractable and the adjacent tissue invasion has not been considered yet. This limitation is more critical for LA-NPC patients, as many vital tissues and organs adjacent to the nasopharynx (e.g., brain, ethmoidal sinus, and orbit) might have already been invaded by LA-NPC [22].

Deep learning is an alternative approach to prognostic prediction and is becoming popular in the literature [15, 23, 24]. Deep survival models based on deep learning usually adopt convolutional neural networks (CNNs) to extract image features and then perform end-to-end prediction from medical images, where tumor segmentation masks are often not required [25]. Without tumor masks as constraints, deep survival models may potentially leverage the prognostic information existing within the entire images. Deep survival models have demonstrated the potential to outperform conventional radiomics-based prognostic prediction models [26–28]. However, performing end-to-end prediction without using tumor masks introduces interference from ﻿non-relevant background information and incurs difficulties in extracting tumor-specific information. Recently, multi-task deep survival models were explored to perform prognostic prediction jointly with tumor segmentation [29–31], which ﻿implicitly guided the model to extract tumor-related information while not discarding ﻿out-of-tumor information. However, the value of multi-task deep learning for prognostic prediction in LA-NPC has not been validated with large patient cohorts. In addition, deep survival models are limited by the ‘block box’ nature [32], which undermines their generalizability in clinical practice.

Nomograms serve as a common tool for guiding individualized treatments as they can simplify complicated prognostic models to numerical estimate of survival probability and provide a clear visual illustration of the factors leading to the prediction [33, 34]. Zhang et al. [5] developed a multiparametric magnetic resonance imaging (MRI)-based radiomic nomogram, which provides an illustrative example of precision medicine and prognostic prediction. Peng et al. [15] developed a deep learning FDG-PET/CT-based nomogram that may act as an individual chemotherapy (IC) indicator in advanced NPC. Pan et al. [3] developed a radiomic nomogram with better prognostic performance than the 8th edition of AJCC/UICC staging system. Nevertheless, it has been reported with an external validation cohort that Pan et al.’s nomogram underestimated the 5-year overall survival (OS) of LA-NPC patients [35]. Therefore, a more reliable and accurate prognostic nomogram is still needed for LA-NPC patients.

In this study, we aim to evaluate the value of multi-task deep learning for prognostic prediction in LA-NPC patients with a large database acquired from two medical centers. We adopted the state-of-the-art deep multi-task survival model (DeepMTS) [29] for joint prognostic prediction and tumor segmentation from pretreatment FDG-PET/CT images, which predicted a survival risk score (DeepMTS-Score) and a tumor segmentation mask for individual LA-NPC patient. The DeepMTS-Score can be directly used for prognostic prediction, while the predicted tumor masks were leveraged for prognostic prediction through radiomics analysis (AutoRadio-Score). We further developed a multi-task deep learning-based radiomic (MTDLR) nomogram by integrating DeepMTS-Score, AutoRadio-Score, and clinical data, so as to improve the accuracy and interpretability of prognostic prediction. Compared with conventional radiomic nomograms, our MTDLR nomogram achieved better prognostic performance and enabled better patient stratification, which demonstrated the potential to facilitate personalized treatment planning.

## Materials and methods

### Patients

Between May 2009 and May 2019, the medical records of 903 NPC patients were collected from Fudan University Shanghai Cancer Center (FUSCC) and Shanghai Proton and Heavy Ion Center (SPHIC). The inclusion criteria are as follows: (1) histologically confirmed LA-NPC (TNM stage III or IVa); (2) received concomitant systemic treatment with intensity modulated radiotherapy (IMRT); (3) underwent pretreatment FDG-PET/CT scans; and (4) available clinical data and FDG-PET/CT images. Patients with previous chemotherapy/radiotherapy or other malignant tumors were excluded. Finally, 652 patients from FUSCC and 234 patients from SPHIC were enrolled in this study. Patients from FUSCC were randomly divided into a training cohort (n = 522) and an internal validation cohort (n = 130) with a 4:1 ratio, while patients from SPHIC (n = 234) were used as an external validation cohort and used merely for evaluation purpose.

After completion of initial treatment, each patient was followed up for every 3 months in the first 2 years, then every 6 months in the third to fifth year, and annually thereafter. The follow-up endpoint of this study is PFS, defined as the time from randomization to the date of disease progression or death from any cause. The median follow-up time is 50 months (ranging from 44 to 120 months) for FUSCC and 49 months (ranging from 44 to 97 months) for SPHIC. FUSCC and SPHIC Ethical Committee approved this retrospective study with informed consent obtained from all enrolled patients.

### PET/CT imaging

FDG-PET/CT images were obtained on a Siemens biograph 16HR PET/CT scanner (Knoxville, Tennessee, USA). FDG-PET/CT data acquisition procedure was detailed in Online Resource.

For quantitative analysis, maximum or mean of standardized uptake value (SUV) normalized to body weight and metabolic tumor volume (MTV) were manually computed for tumor lesions by drawing a 3-dimensional volume of interest (VOI). Meanwhile, total lesion glucose (TLG) was calculated according to the formula: TLG = SUV_mean_ × MTV, where the SUV_mean_ and MTV were recorded at the SUV threshold of 2.5.

### Multi-task deep learning-based radiomics analysis

**Fig. 1:**
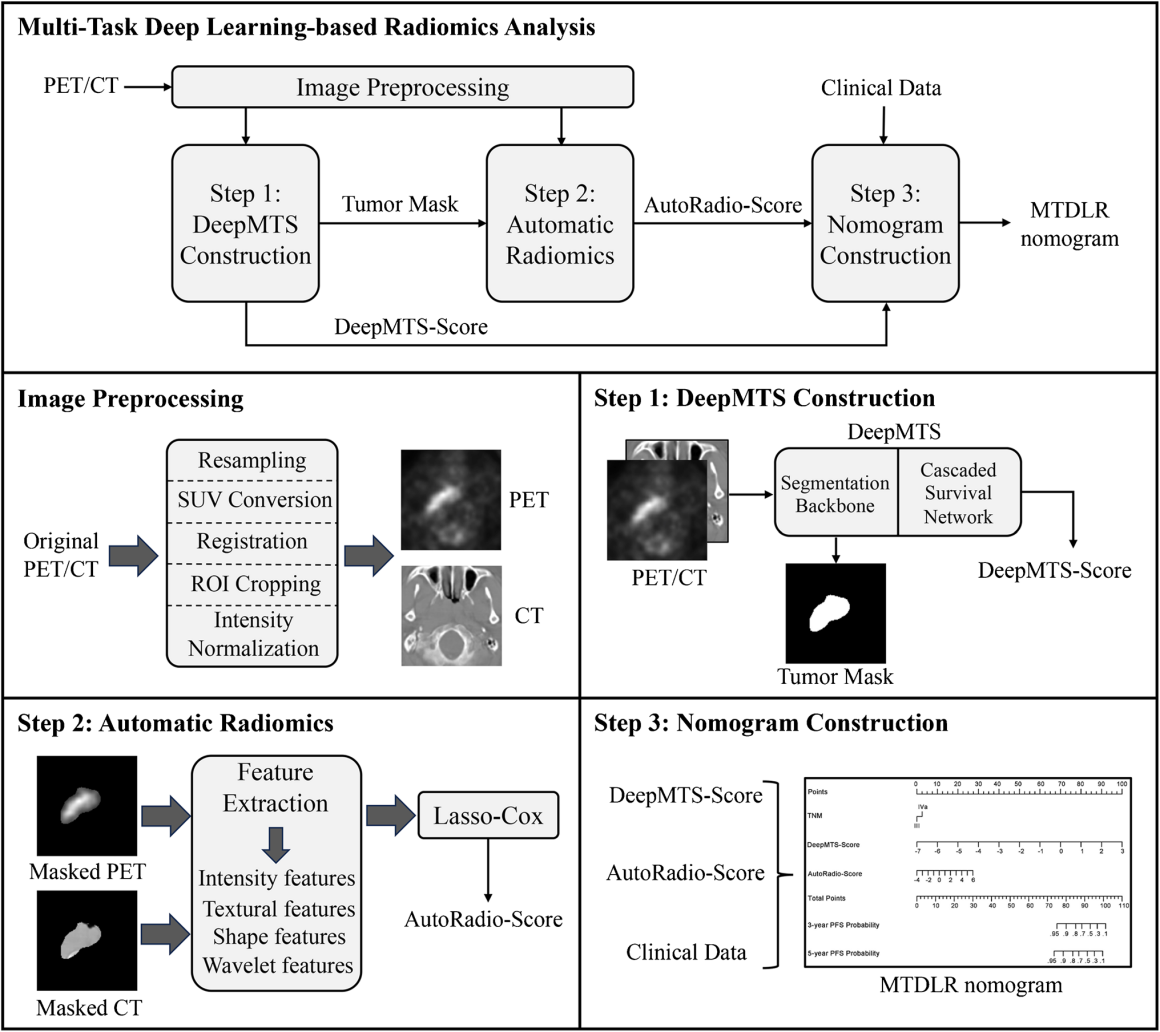
Workflow of multi-task deep learning-based radiomics analysis

The workflow of multi-task deep learning-based radiomics analysis is illustrated in Fig. 1, which presents a three-step pipeline including multi-task deep learning model construction, automatic radiomics analysis, and nomogram construction.

We adopted a deep multi-task survival model (DeepMTS) [29] for joint prognostic prediction and tumor segmentation from FDG-PET/CT images. ﻿We preprocessed FDG-PET/CT images with resampling, SUV conversion (for PET only), affine registration, Regions-of-Interest (ROIs) cropping, and intensity normalization (detailed in Online Resource). The preprocessed PET and CT images were concatenated and fed into the DeepMTS as input, while the manual segmentation masks of primary tumors were used as ground truth labels for training only. The DeepMTS is a CNN ﻿consisting of a Unet-based segmentation backbone [36] and a DenseNet-based cascaded survival network (CSN) [37]. The Unet is a U-shape encoder-decoder CNN with skip connections between its contracting encoder and expanding decoder [36]. The DenseNet is a CNN consisting of multiple dense blocks with dense connections between layers, which enables feature reuse to enhance the capacity to generalize to unseen data [37]. The segmentation backbone is hard-shared by prognostic prediction and tumor segmentation tasks, which implicitly guides the model to extract features related to tumor regions. The outputs of the segmentation backbone are fed into the CSN as a supplementary input (together with FDG-PET/CT images), which further leverages the global tumor information (e.g., tumor size, shape, and locations) for prognostic prediction. Deep features derived from both segmentation backbone and CSN are used for prognostic prediction via two fully-connected layers. After training, DeepMTS can predict the survival risk scores of patients (DeepMTS-Score) and the segmentation masks of tumor regions. The DeepMTS-Score is relevant to PFS and can be directly used for prognostic prediction, while the predicted tumor masks were further leveraged in the following automatic radiomics analysis. The architecture of DeepMTS is detailed in [29] and its implementation code is publicly available at https://github.com/MungoMeng/Survival-DeepMTS. We also provide more training details in Online Resource. For comparison, we also built a single-task deep survival model for prognostic prediction, following Qiang et al.’s study [38], and its output scores are denoted by SingleTask-Score.

With the tumor masks predicted by DeepMTS, ﻿we extracted 1456 handcrafted radiomics features from FDG-PET/CT images via Pyradiomics [39], including 720 PET features, 720 CT features, and 16 shape features based on 3D shape of tumors (detailed in Online Resource). The extracted features were analyzed by a Lasso-Cox model [40], whose output scores are denoted by AutoRadio-Score. We refer to this radiomics process as automatic radiomics, which differentiates it from conventional radiomics based on manual segmentation. For comparison, we also performed the same radiomics analysis based on manual segmentation masks and refer to the output scores as ManualRadio-Score.

After the DeepMTS-Score and AutoRadio-Score are derived, we developed a multi-task deep learning-based radiomic (MTDLR) nomogram by combining the DeepMTS-Score, AutoRadio-Score, and clinical data. Univariate and multivariate analyses were performed for all clinical data and prediction scores via Cox proportional hazards regression, so as to screen out the prognostic indicators with significant relevance to PFS and build the nomogram. For comparison, we also built a conventional radiomic nomogram and a single-task deep learning-based radiomic nomogram by combining the ManualRadio-Score and SingleTask-Score with clinical data.

### Statistical analysis

Continuous parameters were described using median or mean with range, while categorical variables were described using frequency with percentage. Differences among the training, internal validation, and external validation cohorts were analyzed using the Mann–Whitney test, χ^2^ test, or Fisher’s exact test.

Univariate and multivariate Cox analyses were performed using SPSS (version 26.0; IBM Inc., New York, NY, USA). All radiomic nomograms were developed based on the multivariate analyses. Calibration curves with the Hosmer–Lemeshow goodness-of-fit test were applied to evaluate the consistence between the observed PFS proportion and the predicted survival probability.

The prognostic performance of nomograms was evaluated using Harrell's concordance indices (C-index), time-independent receiver operating characteristic (ROC) curve, and area under curve (AUC). The statistical significance between AUCs was tested via DeLong’s method using R packages (version 3.6.3, http://www.R-project.org). Survival analyses based on Kaplan–Meier method were performed for risk group stratification. Patients with score higher/lower than the cutoff value calculated by ROC were stratified into high/low-risk groups, and then a two-sided log-rank test was applied for comparisons. All tests were two-sided for statistical significance, and *P* value < 0.05 was considered to indicate statistically significant differences.

## Results

### Patient characteristics

The demographic and clinical characteristics of patients are presented in Table 1. The median age was 45 years (range 15–83 years), 48 years (range 14–79 years) and 48 years (range 14–74 years) for the training cohort, internal validation cohort, and external validation cohort, respectively. Among these three cohorts, no statistically significant difference was observed in age, gender, EBV DNA, T stage, N stage, and TNM stage, whereas BMI, LDH, histology, and PET parameters were statistically significantly different. At the end of the follow-up, the PFS ratio was 75.67% (395/522), 81.54% (106/130), and 80.77% (189/234) in the training, internal validation, and external validation cohorts, and there was no significant difference of PFS distribution among these cohorts (*P* = 0.163).

**Table 1:** Demographic and clinical characteristics of patients

Characteristics	Training cohort (*n* = 522)	Internal validation cohort (*n* = 130)	External validation cohort (*n* = 234)	*P* value
Age (years), median (range)	45 (15–83)	48 (14–79)	48 (14–74)	0.385
Gender				0.984
Male	402 (77.01%)	101 (77.69%)	180 (76.92%)	
Female	120 (22.99%)	29 (22.31%)	54 (23.08%)	
EBV antibody				0.185
Negative	92 (17.62%)	33 (25.38%)	42 (17.95%)	
Positive	346 (66.28%)	72 (55.38%)	155 (66.24%)	
Unknown	84 (16.10%)	25 (19.24%)	37 (15.81%)	
Histology, WHO Type ^a^				**0.009**
I	4 (0.77%)	2 (1.54%)	2 (0.86%)	
II	50 (9.58%)	14 (10.77%)	6 (2.56%)	
III	468 (89.65%)	114 (87.69%)	226 (96.58%)	
BMI (Kg/m^2^), mean (range)	23.22 (14.69–38.89)	23.35 (15.23–31.41)	24.09 (16.41–34.38)	**0.002**
LDH (U/L), mean (range)	198.02 (89–782)	177.68 (101–728)	212.89 (111–1400)	**0.011**
T stage				0.125
T1	133 (25.48%)	34 (26.15%)	68 (29.06%)	
T2	52 (9.96%)	17 (13.08%)	29 (12.39%)	
T3	289 (55.36%)	65 (50.00%)	128 (54.70%)	
T4	48 (9.20%)	14 (10.77%)	9 (3.85%)	
N stage				0.116
N0	29 (5.56%)	1 (0.77%)	6 (2.56%)	
N1	130 (24.90%)	33 (25.38%)	54 (23.08%)	
N2	301 (57.66%)	76 (58.46%)	137 (58.55%)	
N3	62 (11.88%)	20 (15.39%)	37 (15.81%)	
TNM stage				0.352
III	416 (79.69%)	97 (74.62%)	189 (80.77%)	
IVa	106 (20.31%)	33 (25.38%)	45 (19.23%)	
Concomitant systemic treatment with IMRT				
IC	472 (90.42%)	121 (93.08%)	222 (94.87%)	0.101
CCRT	343 (65.71%)	82 (63.08%)	157 (67.09%)	0.741
AC	98 (18.77%)	24 (18.46%)	42 (17.95%)	0.964
Targeted Therapy	72 (13.79%)	11 (8.46%)	24 (10.26%)	0.151
PET Parameters, mean (range)				
Maximum diameter (cm)	3.63 (0.87–9.81)	3.21 (0.60–6.95)	3.86 (0.82–10.10)	**0.001**
SUV_max_ (g/ml)	12.19 (2.76–33.29)	12.14 (3.36–46.54)	14.82 (2.91–70.84)	** < 0.001**
SUV_mean_ (g/ml)	4.84 (2.60–11.24)	4.77 (2.79–9.23)	5.11 (2.61–18.43)	**0.025**
MTV (ml)	35.70 (0.29–310.48)	33.84 (0.51–199.58)	24.92 (0.18–99.32)	** < 0.001**
TLG (g)	190.64 (0.76–1950.18)	177.06 (1.46–956.73)	144.98 (0.47–1624.22)	**0.005**
PFS				0.163
Progression free	395 (75.67%)	106 (81.54%)	189 (80.77%)	
Progression	127 (24.33%)	24 (18.46%)	45 (19.23%)	

^a^ WHO Type I = keratinizing, WHO Type II = non-keratinizing (differentiated), WHO Type III = non-keratinizing (undifferentiated)

*P* value less than 0.05 was in bold

*EBV* Epstein–Barr virus, *WHO* World Health Organization, *BMI* body mass index, *LDH* lactate dehydrogenase, *IMRT* intensity-modulated radiation therapy, *IC* induction chemotherapy, *CCRT* concurrent chemoradiotherapy, *AC* adjuvant chemotherapy, *SUV* standardized uptake value, *MTV* metabolic tumor volume, *TLG* total lesion glycolysis, *PFS* progression-free survival

### Establishment of MTDLR nomogram

Among the clinical and conventional PET parameters, only TNM stage was significantly associated with PFS in univariate analysis for the training cohort (*P* = 0.031, Table 2). However, none of these parameters showed a significant correlation with PFS in the internal and external validation cohorts. Notably, all the DeepMTS-Score, SingleTask-Score, AutoRadio-Score, and ManualRadio-Score were significantly associated with PFS in univariate analysis for the training, internal and external validation cohorts. For multivariate analysis, the DeepMTS-Score and AutoRadio-Score could serve as independent factors for predicting disease progression in all three cohorts (Table 3).

**Table 2:** Univariate Cox proportional hazard regression analysis for PFS on the training, internal validation, and external validation cohorts

Characteristics	Training cohort		Internal validation cohort		External validation cohort	
	HR (95% CI)	*P* value	HR (95% CI)	*P* value	HR (95% CI)	*P* value
Age	1.006 (0.992–1.019)	0.414	1.029 (0.994–1.065)	0.105	1.004 (0.978–1.031)	0.747
Gender						
Male	Reference	-	Reference	-	Reference	-
Female	0.908 (0.593–1.389)	0.656	1.120 (0.444–2.822)	0.810	0.818 (0.394–1.699)	0.591
EBV antibody	-	0.161	-	0.527	-	0.068
Negative	Reference	-	Reference	-	Reference	-
Positive	0.668 (0.433–1.030)	0.068	1.127 (0.437–2.905)	0.805	2.050 (0.720–5.832)	0.178
Unknown	0.847 (0.487–1.472)	0.556	0.549 (0.136–2.212)	0.399	3.558 (1.145–11.051)	**0.028**
Histology	-	0.399	-	0.992	-	0.960
I	2.483 (0.613–10.052)	0.202	0.000	0.984	0.000	0.977
II	0.879 (0.473–1.632)	0.683	1.079 (0.321–3.627)	0.902	0.749 (0.103–5.441)	0.775
III	Reference	-	Reference	-	Reference	-
BMI	0.978 (0.925–1.033)	0.417	1.083 (0.952–1.233)	0.224	1.041 (0.953–1.137)	0.378
LDH	1.001 (1.000–1.003)	0.113	1.003 (0.999–1.006)	0.114	0.998 (0.995–1.002)	0.321
T stage	-	0.773	-	0.610	-	0.364
T1	Reference	-	Reference	-	Reference	-
T2	0.770 (0.392–1.513)	0.448	1.596 (0.357–7.133)	0.541	0.911 (0.286–2.906)	0.875
T3	0.849 (0.566–1.272)	0.427	2.122 (0.698–6.452)	0.185	1.726 (0.846–3.520)	0.134
T4	1.024 (0.543–1.931)	0.941	1.953 (0.437–8.734)	0.381	0.000	0.969
N stage	-	0.145	-	0.515	-	**0.032**
N0	0.552 (0.223–1.369)	0.200	0.000	0.983	0.396 (0.052–3.027)	0.372
N1	0.512 (0.287–0.915)	**0.024**	1.075 (0.352–3.289)	0.898	0.585 (0.267–1.282)	0.180
N2	0.691 (0.426–1.121)	0.135	0.564 (0.196–1.624)	0.288	0.347 (0.172–0.704)	**0.003**
N3	Reference	-	Reference	-	Reference	-
TNM stage						
III	Reference	-	Reference	-	Reference	-
IVa	1.541 (1.041–2.283)	**0.031**	1.457 (0.624–3.405)	0.385	1.839 (0.965–3.505)	0.064
Maximum diameter	1.082 (0.945–1.238)	0.255	1.136 (0.843–1.531)	0.401	1.054 (0.868–1.280)	0.594
SUV_max_	1.007 (0.973–1.042)	0.688	1.028 (0.976–1.082)	0.297	0.991 (0.955–1.028)	0.626
SUV_mean_	1.036 (0.900–1.193)	0.618	1.109 (0.825–1.490)	0.493	1.000 (0.852–1.175)	0.995
MTV	1.005 (1.000–1.009)	0.054	1.000 (0.987–1.014)	0.968	1.005 (0.991–1.018)	0.504
TLG	1.001 (1.000–1.001)	0.138	1.000 (0.998–1.002)	0.921	1.000 (0.999–1.002)	0.764
ManualRadio-Score	2.304 (1.909–2.781)	** < 0.001**	1.577 (1.136–2.190)	**0.006**	1.703 (1.235–2.348)	**0.001**
SingleTask-Score	4.365 (3.162–6.026)	** < 0.001**	2.669 (1.448–4.919)	**0.002**	1.724 (1.157–2.569)	**0.007**
DeepMTS-Score	5.409 (4.016–7.283)	** < 0.001**	2.633 (1.556–4.457)	** < 0.001**	1.771 (1.219–2.574)	**0.003**
AutoRadio-Score	1.818 (1.616–2.045)	** < 0.001**	2.000 (1.250–3.199)	**0.004**	1.615 (1.208–2.161)	**0.001**

*P* value less than 0.05 was in bold

*PFS* progression-free survival, *HR* hazard ratio, *CI* confidence interval, *EBV* Epstein–Barr virus, *BMI* body mass index, *LDH* lactate dehydrogenase, *SUV* standardized uptake value, *MTV* metabolic tumor volume, *TLG* total lesion glycolysis

**Table 3:** Multivariate Cox proportional hazard regression analysis for PFS on the training, internal validation, and external validation cohorts

Characteristics	Training cohort		Internal validation cohort		External validation cohort	
	HR (95% CI)	*P* value	HR (95% CI)	*P* value	HR (95% CI)	*P* value
TNM stage						
III	Reference	-	Reference	-	Reference	-
IVa	1.464 (0.986–2.174)	0.058	1.229 (0.516–2.929)	0.642	1.786 (0.930–3.432)	0.082
AutoRadio-Score	1.491 (1.314–1.693)	** < 0.001**	1.679 (1.029–2.738)	**0.038**	1.489 (1.102–2.012)	**0.010**
DeepMTS-Score	4.193 (3.074–5.718)	** < 0.001**	2.455 (1.399–4.308)	**0.002**	1.520 (1.037–2.229)	**0.032**

*P* value less than 0.05 was in bold

*PFS* progression-free survival, *HR* hazard ratio, *CI* confidence interval

**Fig. 2:**
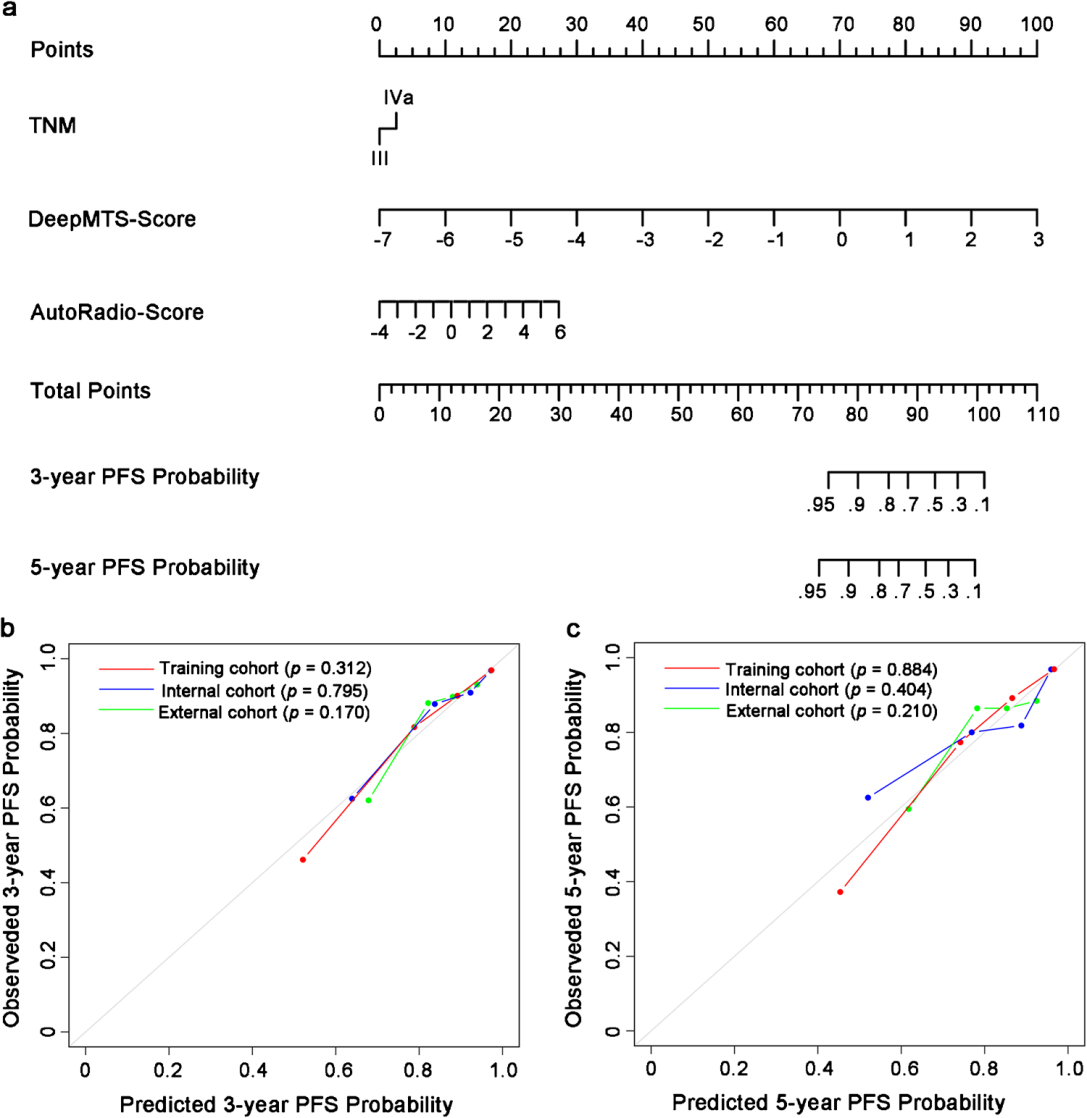
Nomogram and calibration curves. **a** An integrated MTDLR nomogram was built with TNM stage, DeepMTS-derived prognostic prediction score (DeepMTS-Score), and DeepMTS-derived automatic radiomics score (AutoRadio-Score) to predict 3-year and 5-year PFS probability. For calculating the 3-year and 5-year PFS probability with the nomogram, firstly, we locate the patient’s TNM stage and draw a line straight upward to the “Points” axis to determine the points associated with the corresponding TNM stage. Then, we repeat the process for DeepMTS-Score and AutoRadio-Score, and sum the total points achieved for the three covariates. Lastly, we locate this sum on the “Total Points” axis, and draw a line straight down to determine the probability of 3-year and 5-year PFS. **b** The 3-year and **c** 5-year PFS calibration curves of the integrated MTDLR nomogram in the training, internal validation, and external validation cohorts. The actual PFS probability is plotted on the y-axis, while nomogram predicted probability is plotted on the x-axis. The *P* value of calibration was calculated by Hosmer–Lemeshow goodness-of-fit test, and *P* value > 0.05 indicates the good match between the actual and predicted PFS probability

Based on the multivariate analysis, we built the MTDLR nomogram with TNM stage, AutoRadio-Score, and DeepMTS-Score (Fig. 2a). The C-index of the nomogram was 0.818 (95% confidence interval (CI): 0.785–0.851, *P* < 0.001), 0.752 (95% CI: 0.638–0.865, *P* < 0.001), and 0.717 (95% CI: 0.641–0.793, *P* < 0.001) in the training, internal validation, and external validation cohort. Furthermore, the calibration curves showed that the predicted 3-year and 5-year PFS probability of the nomogram was highly consistent with the observed PFS probability (Hosmer–Lemeshow test: *P* > 0.05, Fig. 2b and c).

### Performance of radiomic nomograms

**Fig. 3:**
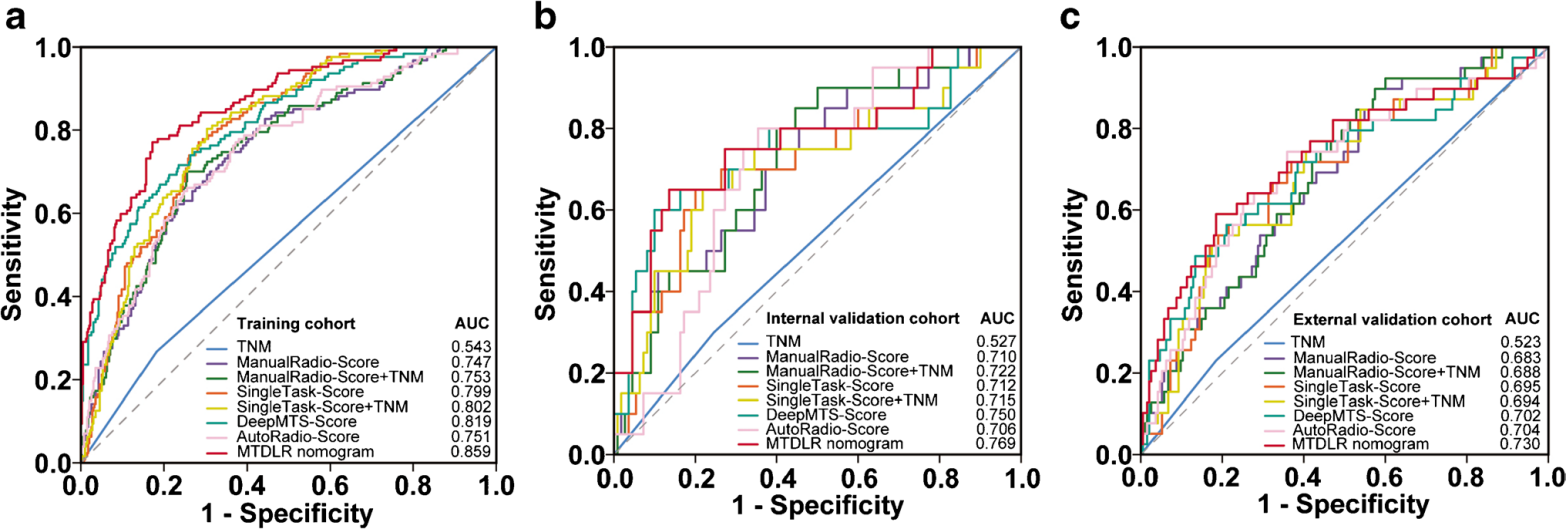
ROC curves for comparison among different clinical, conventional, and deep learning-based radiomics scores/nomograms on the training (**a**), internal validation (**b**), and external validation (**c**) cohorts

To evaluate the prognostic performance of our MTDLR nomogram, the conventional radiomic nomogram (ManualRadio-Score + TNM) and the single-task deep learning-based radiomic nomogram (SingleTask-Score + TNM) were compared (Online Resource Fig. 1 and Table 1). Table 4 shows that our DeepMTS-Score exhibits better prognostic performance than the SingleTask-Score in the training (C-index and AUC: 0.780 and 0.819; Fig. 3a), internal validation (0.731 and 0.750; Fig. 3b), and external validation cohorts (0.695 and 0.702; Fig. 3c). Furthermore, the AutoRadio-Score also shows better prognostic performance than the ManualRadio-Score in these three cohorts (C-index: 0.728, 0.702, and 0.669; AUC: 0.751, 0.706, and 0.704). Moreover, the MTDLR nomogram combining TNM stage, DeepMTS-Score, and AutoRadio-Score achieved the best prognostic performance among all prognostic scores and nomograms in all three cohorts (C-index: 0.818, 0.752, and 0.717; AUC: 0.859, 0.769, and 0.730).

**Table 4:** C-index and AUC of different clinical, conventional, and deep learning-based radiomic scores/nomograms evaluated on the training, internal validation, and external validation cohorts

Signatures	C-index (95% CI)	AUC (95% CI)	*P* value			
			C-index	Com1*	AUC	Com2*
Training cohort						
TNM	0.538 (0.500–0.576)	0.543 (0.500–0.586)	0.028	Reference	0.981	Reference
ManualRadio-Score	0.720 (0.678–0.762)	0.747 (0.698–0.795)	< 0.001	0.015	< 0.001	< 0.001
ManualRadio-Score + TNM	0.725 (0.683–0.767)	0.753 (0.705–0.801)	< 0.001	0.008	< 0.001	< 0.001
SingleTask-Score	0.767 (0.731–0.803)	0.799 (0.759–0.839)	< 0.001	< 0.001	< 0.001	< 0.001
SingleTask-Score + TNM	0.770 (0.734–0.806)	0.802 (0.762–0.842)	< 0.001	< 0.001	< 0.001	< 0.001
DeepMTS-Score	0.780 (0.741–0.819)	0.819 (0.777–0.861)	< 0.001	< 0.001	< 0.001	< 0.001
AutoRadio-Score	0.728 (0.685–0.771)	0.751 (0.703–0.799)	< 0.001	0.009	< 0.001	< 0.001
MTDLR nomogram	**0.818 (0.785–0.851)**	**0.859 (0.822–0.895)**	< 0.001	< 0.001	< 0.001	< 0.001
Internal validation cohort						
TNM	0.526 (0.435–0.617)	0.527 (0.417–0.638)	0.578	Reference	0.698	Reference
ManualRadio-Score	0.680 (0.578–0.782)	0.710 (0.587–0.833)	< 0.001	0.049	0.001	0.051
ManualRadio-Score + TNM	0.693 (0.595–0.791)	0.722 (0.606–0.839)	< 0.001	0.016	0.001	0.019
SingleTask-Score	0.705 (0.587–0.823)	0.712 (0.577–0.846)	< 0.001	0.005	0.001	0.014
SingleTask-Score + TNM	0.708 (0.589–0.827)	0.715 (0.579–0.851)	< 0.001	0.002	0.001	0.008
DeepMTS-Score	0.731 (0.605–0.856)	0.750 (0.609–0.890)	< 0.001	0.009	< 0.001	0.011
AutoRadio-Score	0.702 (0.619–0.785)	0.706 (0.597–0.815)	< 0.001	0.015	0.002	0.021
MTDLR nomogram	**0.752 (0.638–0.865)**	**0.769 (0.642–0.896)**	< 0.001	0.001	< 0.001	0.002
External validation cohort						
TNM	0.554 (0.489–0.619)	0.523 (0.451–0.595)	0.628	Reference	0.595	Reference
ManualRadio-Score	0.642 (0.567–0.717)	0.683 (0.596–0.770)	< 0.001	0.111	< 0.001	0.009
ManualRadio-Score + TNM	0.655 (0.582–0.728)	0.688 (0.603–0.773)	< 0.001	0.039	< 0.001	0.003
SingleTask-Score	0.655 (0.575–0.735)	0.695 (0.603–0.786)	< 0.001	0.056	< 0.001	0.002
SingleTask-Score + TNM	0.662 (0.582–0.742)	0.694 (0.602–0.786)	< 0.001	0.024	< 0.001	0.001
DeepMTS-Score	0.695 (0.616–0.774)	0.702 (0.603–0.801)	< 0.001	0.033	< 0.001	0.001
AutoRadio-Score	0.669 (0.589–0.749)	0.704 (0.609–0.799)	< 0.001	0.109	< 0.001	0.004
MTDLR nomogram	**0.717 (0.641–0.793)**	**0.730 (0.634–0.826)**	< 0.001	0.009	< 0.001	< 0.001

^*^Com1 means the *P* value was for the comparison of C-index, and Com2 was for AUC

The best result in each cohort was in bold

*AUC* area under the curve, *CI* confidence interval

### Survival analysis for risk group stratification

**Fig. 4:**
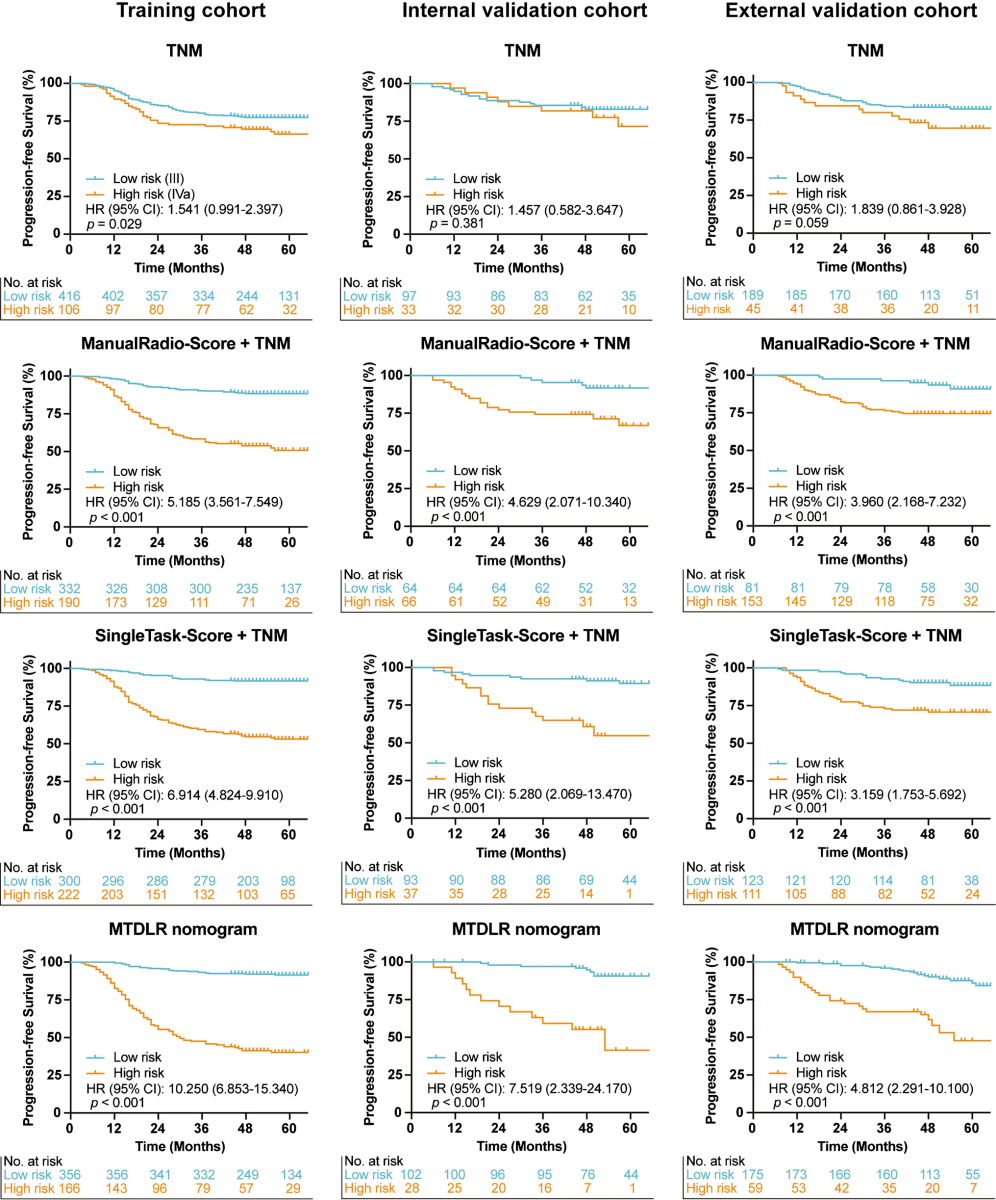
Kaplan–Meier curves of risk group stratification based on TNM stage, ManualRadio-Score + TNM, SingleTask-Score + TNM, and MTDLR nomogram on the training, internal validation, and external validation cohorts

The conventional radiomic nomogram (ManualRadio-Score + TNM), single-task deep learning-based radiomic nomogram (SingleTask-Score + TNM), and our MTDLR nomogram were used to stratify patients into high- and low-risk groups by cutoff values calculated with ROC curves. The Kaplan–Meier curves of the high- and low-risk patient groups were showed in Fig. 4. For comparison, the commonly-used TNM stage was also adopted to stratify patients according to stage III or IVa, where the patients with stage IVa had significantly poorer prognosis than the patients with stage III in the training cohort (Hazard rate (HR): 1.541, 95% CI: 0.991–2.397, *P* = 0.029). However, the TNM stage failed to stratify patients into significantly different groups in the internal and external validation cohorts (HR: 1.457, 95% CI: 0.582–3.647, *P* = 0.381 and HR: 1.839, 95% CI: 0.861–3.928, *P* = 0.059, respectively). Figure 4 also show that all three nomograms stratify patients into significantly different groups in all three cohorts (*P* < 0.001). Nevertheless, our MTDLR nomogram differentiated the high- and low-risk groups with the highest HR value among these three nomograms (HR: 10.250, 95% CI: 6.853–15.340, in the training cohort; HR: 7.519, 95% CI: 2.339–24.170, in the internal validation cohort; and HR: 4.812, 95% CI: 2.291–10.100, in the external validation cohort). In addition, the Kaplan–Meier curves of the patient groups stratified by ManualRadio-Score, SingleTask-Score, DeepMTS-Score, and AutoRadio-Score were presented in Online Resource Fig. 2.

## Discussion

In this study, we constructed a multi-task deep learning-based radiomic (MTDLR) nomogram to predict the PFS of LA-NPC patients. The prognostic prediction and risk stratification performance of the MTDLR nomogram was superior to the conventional radiomic nomogram and single-task deep learning-based radiomic nomogram. LA-NPC patients can be stratified into low- and high-risk groups, where the high-risk group was characterized by worse PFS rates than the low-risk group.

The TNM staging system, focusing on anatomical and locational information, has been widely used in clinical studies [7–9] but, unfortunately, was not an independent prognostic factor in our study (Table 3). Nevertheless, we identified that combining TNM stage with other prognostic scores still improved the prognostic performance, which is consistent with the findings reported in previous studies [8, 28, 35]. FDG-PET/CT images, given the capabilities in providing tumors’ metabolic and anatomical information, have also been widely used for prognostic prediction [41–43]. However, the conventional FDG-PET/CT-derived parameters (SUV, MTV, and TLG) cannot serve as effective prognostic indicators in our univariate analysis (Table 2). To further leverage the prognostic information in FDG-PET/CT images, radiomics or deep learning were adopted and showed superiority over conventional parameters [28, 44]. Nevertheless, the prognostic performance varied with different radiomics or deep learning models, which suggests that the prognostic information in FDG-PET/CT image cannot be easily accessed and should be carefully leveraged with well-developed models.

Currently, there is a dilemma for extracting prognostic information from medical images. As discussed, conventional radiomics can well characterize the intra-tumor information while it is limited to the segmented tumor regions. Deep learning can access the prognostic information in the entire images. However, it has difficulties in extracting tumor-specific information. In this study, we adopted a deep multi-task survival model (DeepMTS) [29] to address this dilemma. It has been demonstrated that, through jointly learning tumor segmentation task with a hybrid multi-task architecture, DeepMTS can effectively extract ﻿prognostic information from tumor regions while also capturing the out-of-tumor prognostic information, which enables DeepMTS to outperform existing radiomics- or deep learning-based prognostic prediction models [29]. Nevertheless, we noticed that the segmentation output of DeepMTS was not fully leveraged for prognostic prediction and the prognostic information within tumor regions could be further explored. Therefore, we used the DeepMTS-segmented tumor masks for automatic radiomics analysis, which further explored the intra-tumor prognostic information and removed the reliance of conventional radiomics on manual segmentation. For tumor segmentation, the DeepMTS achieved a Dice Similarity Coefficient (DSC) of 0.826, 0.775, and 0.765 on the training, internal validation, and external validation cohorts, which demonstrates great consistency with the manually delineated segmentation masks. It has been reported that automatic segmentations improved the objectiveness [45] and resulted in significantly better prognostic prediction performance than manual segmentation [46], which potentially enables better radiomics analysis and facilitates the final prognostic prediction [47, 48].

The prognostic scores from DeepMTS and automatic radiomics were combined with clinical data to build the MTDLR nomogram, which leveraged both FDG-PET/CT and clinical information and also improved the interpretability for prediction. Our MTDLR nomogram achieved the best prognostic performance among all comparison prognostic scores and nomograms (Table 4), which could be attributed to three facts. First, the DeepMTS produced more discriminative prognostic scores (DeepMTS-Score) than the commonly used single-task deep survival model (SingleTask-Score). Second, the automatic radiomics also produced more discriminative prognostic scores (AutoRadio-Score) than conventional radiomics (ManualRadio-Score). Finally, the DeepMTS-Score and AutoRadio-Score were combined together to achieve better prognostic prediction. The strategy of combining multi-task deep learning and radiomics has been adopted for prognostic prediction in head and neck cancer [48] and achieved one of the top prognostic performance in HEad and neCK TumOR segmentation and outcome prediction (HECKTOR 2022) challenge [49]. Our study further validated this strategy with a large database of NPC patients.

We divided patients based on our MTDLR nomogram and found that the MTDLR nomogram effectively stratified LA-NPC patients into significantly different risk groups, which is potentially beneficial for individualized treatment regimens. Induction chemotherapy (IC) plus concurrent chemoradiotherapy (CCRT) is recommended as 2A-level evidence according to the National Comprehensive Cancer Network (NCCN) guidelines [4]. However, it’s still a controversy as a portion of LA-NPC patients do not benefit from IC. Qiang et al. [38] developed a prognostic system to explore whether high-risk or low-risk patients can benefit from IC + CCRT than CCRT only. Zhong et al. [16] developed a deep learning-based radiomic nomogram to predict the prognosis of NPC patients with different regimens and accordingly recommend an optimal treatment regimen. These studies demonstrated the necessity of stratifying LA-NPC patients into different risk groups so as to optimize treatment regimens.

There exist several inevitable limitations with our study. First, the completeness and homogeneity of our data had deficiencies due to its retrospective nature. EBV status was missing for about 15% of patients, which might limit the accuracy of statistical analysis. Second, our study was conducted in endemic areas and thus only included patients with TNM stage III and IVa. Therefore, the MTDLR nomogram could be further validated with more extensive databases in future studies. However, it should be noted that we have validated our MTDLR nomogram in a large database (886 patients) with two validation cohorts, which can support the effectiveness of MTDLR nomogram in LA-NPC.

## Conclusion

In this study, we evaluated the value of multi-task learning for prognostic prediction in LA-NPC patients. To achieve this, we adopted a deep multi-task survival model (DeepMTS) and developed a multi-task deep learning-based radiomic (MTDLR) nomogram that combines TNM stage, DeepMTS-Score, and AutoRadio-Score. Compared to the conventional and single-task deep learning-based radiomic nomograms, the MTDLR nomogram extracted more heterogeneous and prognostic information to better predict the prognosis of LA-NPC patients. We validated our MTDLR nomogram with a large LA-NPC databased with two (internal/external) validation cohorts, which support the effectiveness of MTDLR nomogram and its potential contributions to clinical decision making.

### Supplementary Information

Below is the link to the electronic supplementary material.

Supplementary file1 (DOCX 883 KB)

## Data Availability

Data generated or analyzed during the study are available from the corresponding author by request.
