# Supplementary material for: Multi-task deep learning-based radiomic nomogram for prognostic prediction in locoregionally advanced nasopharyngeal carcinoma
Source: Eur J Nucl Med Mol Imaging. 2023 Aug 19;50(13):3996–4009. doi: 10.1007/s00259-023-06399-7 (PMC10611876; doi:10.1007/s00259-023-06399-7)
Supplement: Supplementary file 1 — Supplementary file1 (DOCX 883 KB) [file 259_2023_6399_MOESM1_ESM.docx]

**Multi-task deep learning-based radiomic nomogram for prognostic prediction in locoregionally advanced nasopharyngeal carcinoma**

Bingxin Gu^1^ • Mingyuan Meng^2^ • Mingzhen Xu^1^ • David Dagan Feng^2^ •Lei Bi^3^ • Jinman Kim^2^ • Shaoli Song^1^

^1^ Department of Nuclear Medicine, Fudan University Shanghai Cancer Center; Department of Oncology, Shanghai Medical College, Fudan University; Center for Biomedical Imaging, Fudan University; Shanghai Engineering Research Center of Molecular Imaging Probes; Key Laboratory of Nuclear Physics and Ion-beam Application (MOE), Fudan University; Shanghai, PR China.

^2^ School of Computer Science, the University of Sydney, Sydney, Australia.

^3^ Institute of Translational Medicine, National Center for Translational Medicine, Shanghai Jiao Tong University, Shanghai, China.

**Bingxin Gu, Mingyuan Meng, and Mingzhen Xu contributed equally to this work.**

**Correspondence to:**

**Shaoli Song,** email: shaoli-song@163.com

**Jinman Kim,** email: jinman.kim@sydney.edu.au

**Lei Bi,** email: lei.bi@sjtu.edu.cn

**Section A. PET/CT imaging**

Patients fasted at least 4 hours to maintain the venous blood glucose levels under 10 mmol/L before injection. Each patient got injected with 7.4 MBq/kg [^18^F]FDG, then kept lying comfortably in a quiet, dimly lit room for approximately 1 hour prior to scanning. PET/CT data acquisition procedure was as follows: CT scanning was first performed, from the proximal thighs to head, with 120 kV, 80 ~ 250 mA, pitch 3.6, rotation time 0.5, slice thickness: 5.0 mm. Immediately after CT scanning, a PET emission scan that covered the identical transverse field of view was obtained. Acquisition time was 2 ~ 3 min per table position. PET images were reconstructed iteratively using an ordered-subset expectation maximization iterative reconstruction (OSEM) by applying CT images for attenuation correction. The reconstruction parameters were as follows: iterations: 4, subsets: 8, pixel size: 4.0 × 4.0 mm, zoom: 1.0, FWHM: 6.0 mm, and slice thickness: 5.0 mm. Fusion images were reviewed and manipulated on a multimodality computer platform (Syngo, Siemens, Knoxville, Tennessee, USA).

**Section B. FDG-PET/CT image preprocessing**

FDG-PET/CT images were preprocessed through the following steps: (1) Tumor masks were segmented on PET and CT images simultaneously by an experienced nuclear medicine physician (B.G., with > 10 years of experience in FDG-PET/CT), using a semi-automatic segmentation algorithm available in ITK-SNAP software (version 3.8.0, <http://www.itksnap.org>). The semi-automatic segmentation masks were then manually adjusted and refined, also using ITK-SNAP, by a senior nuclear medicine physician (S.S., with > 15 years of experience in FDG-PET/CT) to ensure reliability of the segmented masks. (2) FDG-PET/CT images were resampled into isotropic voxels of unit dimension to ensure comparability, where 1 voxel corresponds to 1 mm^3^. (3) PET images were normalized based on body mass. The derived body mass was applied to convert PET images into SUV maps. (4) All images were registered and located in a relatively fixed position. Specifically, we adopted the standard affine registration method implemented in the ANTs package (https://antspyx.readthedocs.io/), which is a conventional optimization-based registration method that maximizes the image similarity through iterative optimization. We performed this method to register the CT images of two patients and then applied the resulted affine transformation matrix to the corresponding PET images and tumor masks. We randomly chose a patient as the template and registered all other patients with it, so that all images were located in a relatively fixed position. (5) The registered FDG-PET/CT images were cropped into 128×128×128 Regions-of-Interest (ROIs) by fixed-position cropping. The fixed cropping positions can be manually decided to cover the whole nasopharynx because all images had been registered and located in a fixed position. (6) PET ROIs were standardized individually to zero mean and unit variance (Z-score normalization), while CT ROIs were clipped to range [0, 2048] and then mapped to range [−1, 1].

**Section C. DeepMTS training details**

The DeepMTS is open source at <https://github.com/MungoMeng/Survival-DeepMTS> and we trained it using Keras with a Tensorflow backend on two 12 GB Titan X GPUs. We used an ﻿Adam optimizer with a batch size of 8 to train the DeepMTS for 15000 iterations. The learning rate was 1e^-4^ initially and then decreased to 5e^-5^, 1e^-5^, and 1e^-6^ at the 2500th, 5000th, and 10000th training iteration. The model was trained in the training cohort and internally validated in the internal validation cohort. Internal validation was performed after every 200 training iterations and the model achieving the highest validation result was preserved for as the final model. During training, data augmentation (random affine transformations) was applied to the input FDG-PET/CT images in real-time to avoid overfitting. We also sampled an equal number of censored and uncensored samples in each mini-batch during data augmentation.

**Section D. Radiomics feature extraction**

With the tumor masks predicted by DeepMTS, we extracted a total of 1456 handcrafted radiomics features from FDG-PET/CT images via Pyradiomics. The extracted radiomics features include 19 features from First Order Statistics (FOS), 24 features from Grey-Level Cooccurrence Matrix (GLCM), 16 features from Grey-Level Run Length Matrix (GLRLM), 16 features from Grey-Level Size Zone Matrix (GLSZM), 5 features from Neighboring Grey Tone Difference Matrix (NGTDM), and 16 features based on 3D tumor shape. The 16 shape-based features were extracted from the tumor segmentation masks, while the other (19+24+16+16+5)=80 features were extracted from PET and CT images separately. The PET/CT-derived features were also recomputed for different wavelet decomposition of PET/CT images. Performing low-pass or high-pass wavelet filter along x, y, or z directions resulted in 8 decompositions (LLL, LLH, LHL, LHH, HHH, HLL, HHL, and HLH). Consequently, we derived a total of 80×(1+8)×2+16=1456 radiomics features. All radiomics features were standardized using Z-score normalization and the redundant features with Spearman’s correlation >0.7 were eliminated.

**Supplementary Table 1.** Multivariate Cox proportional hazard regression analysis for PFS on the training, internal validation, and external validation cohorts

| **Characteristics** | **Training cohort** | |  | **Internal validation cohort** | |  | **External validation cohort** | |
| --- | --- | --- | --- | --- | --- | --- | --- | --- |
|  | **HR (95% CI)** | ***P* value** |  | **HR (95% CI)** | ***P* value** |  | **HR (95% CI)** | ***P* value** |
| TNM stage |  |  |  |  |  |  |  |  |
| III | Reference | - |  | Reference | - |  | Reference | - |
| IVa | 1.406 (0.949-2.084) | 0.089 |  | 1.443 (0.617-3.373) | 0.398 |  | 1.803 (0.946-3.436) | 0.073 |
| ManualRadio-Score | 2.292 (1.898-2.768) | **<0.001** |  | 1.575 (1.133-2.188) | **0.007** |  | 1.714 (1.235-2.379) | **0.001** |
| TNM stage |  |  |  |  |  |  |  |  |
| III | Reference | - |  | Reference | - |  | Reference | - |
| IVa | 1.478 (0.998-2.190) | 0.051 |  | 1.302 (0.554-3.060) | 0.545 |  | 1.742 (0.914-3.320) | 0.092 |
| SingleTask-Score | 4.403 (3.177-6.100) | **<0.001** |  | 2.620 (1.421-4.831) | **0.002** |  | 1.713 (1.144-2.566) | **0.009** |

Note: *P* value less than 0.05 was in bold.

*PFS, progression-free survival; HR, hazard ratio; CI, confidence interval.*

**
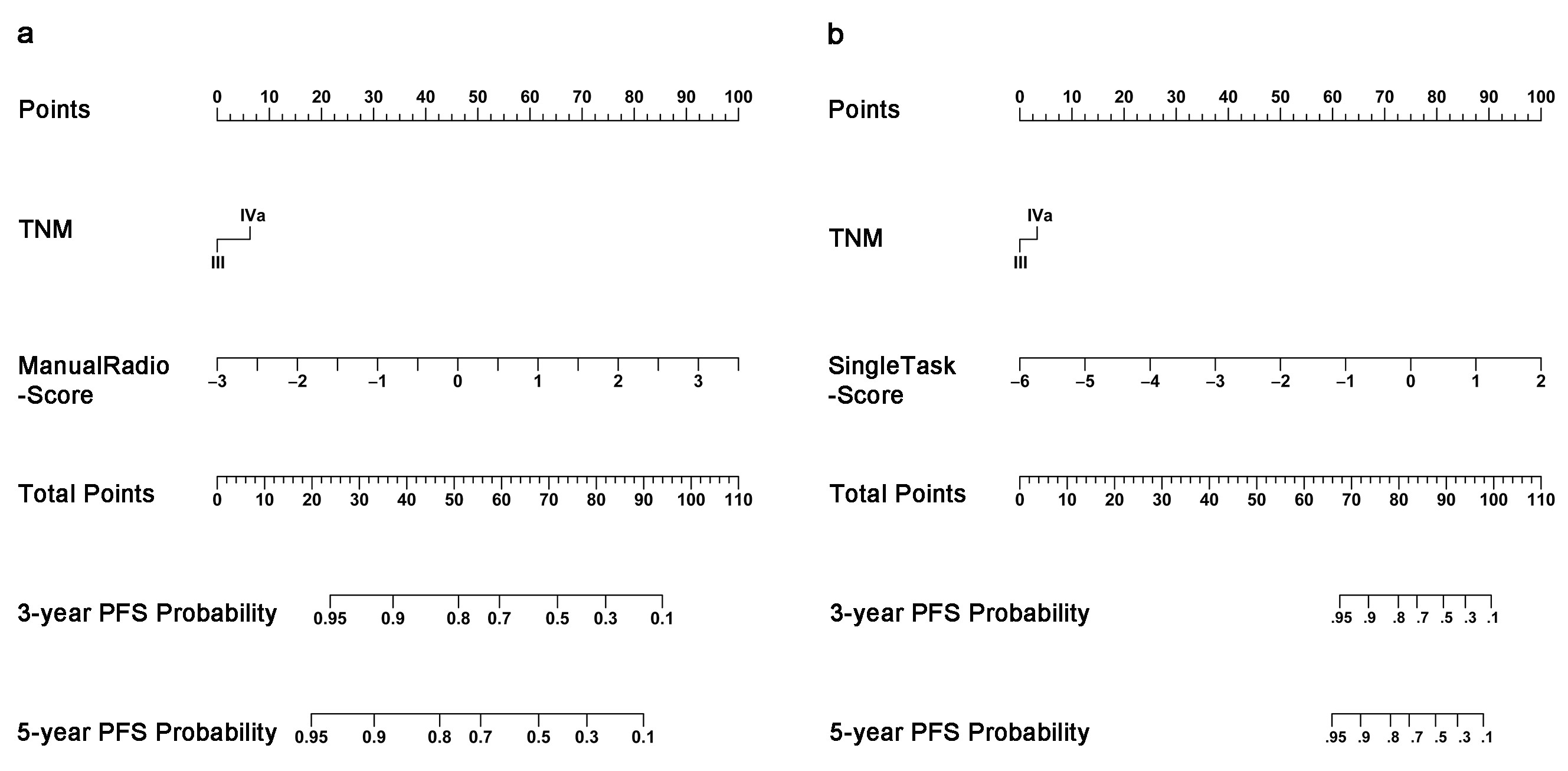
Supplementary Fig. 1** Nomograms built with TNM stage and ManualRadio-Score (a) and with TNM stage and SingleTask-Score (b) to predict 3-year and 5-year PFS.

**
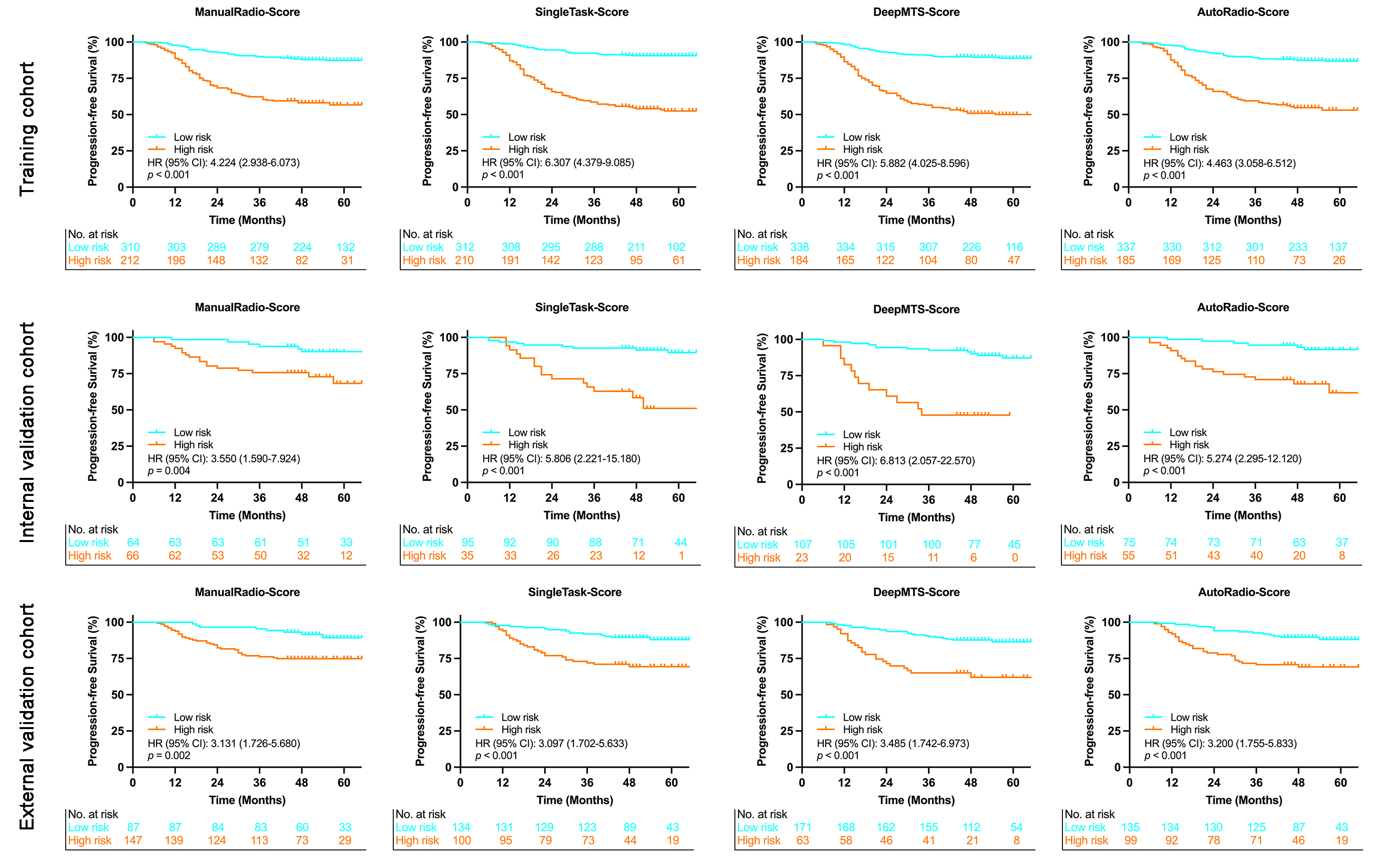
Supplementary Fig. 2** Kaplan-Meier curves of risk group stratification based on ManualRadio-Score, SingleTask-Score, DeepMTS-Score, and AutoRadio-Score on the training, internal validation, and external validation cohorts.
